# LATEX sensitization in elderly: allergological study and diagnostic protocol

**DOI:** 10.1186/1742-4933-11-7

**Published:** 2014-05-02

**Authors:** Teresa Grieco, Valentina Faina, Laura Dies, Marzio Milana, Emidio Silvestri, Stefano Calvieri

**Affiliations:** 1 Department of Dermatology, University of Rome “Sapienza”, Viale del Policlinico, 155, 00161 Rome, Italy

**Keywords:** Allergological study, Elderly, Latex allergy

## Abstract

**Background:**

The prevalence of latex allergy varies according to the population studied from 3% to 64%. No data exist in the present literature about elderly people because they were not considered among populations at risk. We report a retrospective observational study of 88 elderly patients of our centre of Dermatology and Allergology at Policlinico Umberto I, University of Rome, Sapienza.

**Results:**

First and second level diagnostic tests showed latex positivity in 11,4% of patients studied for latex allergy in the elderly population.

**Conclusions:**

Our study demonstrates a prevalence of elderly-latex sensitization of 11,4%, showing that allergy to latex is a growing disease that can occur at any age. So, we propose these patients as an additional risk category for latex allergy.

## Background

Natural rubber latex (NRL) is a natural sap of the rubber tree (*Hevea brasiliensis*), which grows in Africa and the Malaysian peninsular [1,2]. NRL coagulates on exposure to air, giving rise to spherical polyisoprene droplets coated with a layer of water soluble proteins [3]. This compound is filtered and preserved with one of the following: 1) sodium sulfite; 2) formaldehyde; 3) ammonia (0.05–2.0%); or 4) ammonia and 0.025% of a 1:1 mixture of zinc oxide and tetramethylthiuram disulfide in order to alkalinize the pH, which increases stability of NRL and slows down the growth of microorganisms [4-7].

Latex allergy may develop through two major pathways: a) one that is dependent on sensitization to latex protein with a type I immunoglobulin E (IgE)-mediated hypersensitivity reaction, b) one that depends on chemicals mixed with the latex protein such as thiurams, mercapto-benzathiazoles, that are the cause of type IV delayed hypersensitivity reactions [8].

The prevalence of latex allergy varies according the population studied from 3% to 64%, being highest in groups such as healthcare workers, rubber industry workers, patients who have had multiple surgeries, and children with bladder extrophy or myelodysplasia. It is estimated that more than 15 million people suffer from latex allergy worldwide [9-11]. Among the general population the range of sensitization is between 5% and 10%, while in the healthcare workers it is approximately 10 to 17% [12]. Allergy risk is increased in individuals who have cumulatively prolonged exposure to latex for several reasons, such as exposure at work, in the home environment and while engaging in hobbies.

Although latex allergy is most common in adults, no data are available in the present literature on the incidence in elderly people, essentially because they were not considered as risk-population. However, we have clinical evidence that after latex contact, this group of patients may develop severe reactions. Therefore, we report a retrospective observational study of elderly population arrived at our Centre of Dermatology and Allergology at the Policlinico “Umberto I” Hospital of the University of Rome, Sapienza.

The present study aims to determine:

– latex allergy prevalence in elderly people

– the peak of prevalence in relation to sex and age

– class sensitization in our patients

– diseases related to latex allergy

– diseases due to latex allergy in our population of patients.

### Patients and methods

The study "latex sensitization in elderly: allergological study and diagnostic protocol. Retrospective study." was approved by the ethics committee of the University of Rome “Sapienza” (3089/13/02/2014).

#### Study population

From January 2003 to May 2012, 912 patients joined the investigation of first level (RAST) for latex sensitization. Of these 912 patients we selected, on the basis of old age, 88 aged 65 or over, representing our cases study. In particular, we selected 27 males and 61 females, aged from 65 to 86 years whose average age was 71,7 years.

The patients were subjected to the following study protocol:

– clinical history according to American Latex Allergy Association test [13];

– cutaneous clinical examination;

– first level blood tests, according to good practice referred to in the literature [14,15], although there are not any real guidelines:

•RAST test to detect latex-specific IgE;

•specific IgE recognition patterns to recombinant Hevea brasiliensis (Hev b).

– If RAST was negative, second level in vivo tests were performed:

•latex-skin prick test;

•patch test in line with European standard series;

•rubber additional series;

•NRL additional series;

•latex specific series “TRP-LTX 960”;

•patch by patch with latex gloves.

– Challenge tests are not performed for severe adverse reactions risk referred to in the literature.

#### Clinical history

Patients were asked to collect the following information:

– demographic data (name, age, gender, employment status);

– work-related data and after-work activities;

– number of surgical interventions undergone;

– previous respiratory disease;

– family and personal history of atopy;

– cutaneous, nasal, ocular or respiratory symptoms and their association with the workplace and/or with the use of latex gloves or with some hobbies;

– allergic reactions after contact with latex products or with fruits related to latex allergy (banana, kiwi, chestnut, and avocado);

– use of latex gloves or other materials;

– previous diagnosis of latex allergy.

The same data can also be collected by submitting a questionnaire to the patient and the questionnaire from the American Latex Allergy Association was quite helpful [13].

#### Rast and protein microarray test procedure

We used the ImmunoCAP system to determine serum specific IgE to latex and its main allergens in recombinant form (rHev b 1, rHev b 6.01, rHev b 6.02, rHev b 6.03, rHev b 8 and rHev b 11). A reading of more than 0.35 kUA/L was considered positive. In particular, distinguish 7 sensitization classes: class 0 (<0.35 kUA/L), class I (0,35-0,70 kUA/L), class II (0,71-3,50 kUA/L), class III (3,51-17,50 kUA/L), class IV (17,51-50 kUA/L), class V (51-100 kUA/L), class VI (>100 kUA/L).

#### Skin prick test

The skin test procedures are performed with a lancet pricking the skin on the forearm through a drop of latex extracts (ALK-ABELLO’®) at sequential concentrations of 0.001-1 mg/mL of latex protein. The results are read after 15 minutes and are compared to a negative saline control and histamine. The presence of a wheal with an average diameter equal to or greater than 3 mm [16] was interpreted as a positive reaction.

#### Patch test

The allergens are applied to Finn chambers. The Finn chambers are placed on the upper back and removed after 72 hours (D3) for European and rubber series (MERK®). While reading for latex patch test were performed after 30 minutes, 48 hours (D2) and 96 hours (D4). A commercial non-ammoniated latex (ALK-ABELLO®) was used. Positive reactions were evaluated according to International Contact Dermatitis Research Group recommendations [17]. Irritant reactions were considered negative. The patients were also tested with a patch by patch with a latex glove and were also studied with a latex specific series “TRP-LTX 960” (EUROMEDICAL®).

#### Statistical analysis

The cases considered was analyzed statistically with descriptive statistical index (percentage, average). In particular it was calculated the prevalence of subjects with latex-RAST test and latex-SPT positivity. This prevalence was calculated at 95% confidence interval with the Wilson method.

The association between latex sensitization and gender and age, as well as with certain disorders (eg, urticaria) was carried out using the chi square test of Pearson or the Fish’s exact test where appropriate. The level of the first type error (α) has been set equal to 0.05.

## Results

From a total of 88 (27 male, 61 female) elderly patients, aged from 65 to 86 years whose average age was 71,7, 46 of them (52,3%) displayed only cutaneous symptoms:

•irritant/allergic contact dermatitis

•generalized urticaria

•angioedema;

12 patients (13,6%) reported respiratory and/or mucosal reactions

•rhinoconjunctivitis

•asthma;

2 patients (2,2%) reported severe generalized reaction

•glottis oedema

•anaphylactic shock;

3 patients (3,4%) reported an adverse reaction during surgery;

1 patient (1,2%) reported adverse drug reactions;

24 patients (27,3%) showed polisensitivity for aeroallergens such as dermatophagoides, grass pollen and tree pollen.

First level diagnostic test such as latex-RAST test, showed latex positivity in 8 patients, 4 males and 4 females.

Four subjects had a RAST value of I class, 3 of II class and only 1 of III class, with a mean level of 3,5 kUA/L (0.35-100 kUA/L).

In particular, 4 of these positive patients were aged in the range from 75 to 79 years, 2 patients were aged in the range from 65 to 69 years and the other 2 patients in the range from 80 to 84 years with a peak of prevalence from 75 to 79 years.

Microarray-based assessment of serum specific-IgE recognition patterns detected a positive results for the following NRL proteins: 2 patients were rHev b 8 and only 1 patients was rHev b 11 positive.

The second level in vivo investigation tests were performed on 80 serum specific-IgE negative patients. The skin prick test revealed 2 more females with positivity, for a total of 10 patients positive to latex, with a prevalence of 11.4% evaluable in population with a 95% confidence interval between 6.3% and 19.7%. This prevalence is not influenced neither by sex nor age since there is no a significant difference between these variables. In fact, using the chi square test of Pearson or the Fish’s exact test, we have linked the latex sensitization with different diagnoses and sex of patients, but because of the sample is too small, has not revealed any significant results.

Although there are not significant differences regard to diagnosis, we found a higher percentage of sensitization among patients who reported urticaria and angioedema, followed by adverse drug reactions (ADR), contact dermatitis and finally asthma and polisensitivity to aeroallergens.

All patients were also patch tested according to European standard series, NRL, additional rubber series, patch by patch with a latex gloves, and with a latex specific series “TRP-LTX 960” (EUROMEDICAL®).

The European series showed 1 positivity for nickel sulphate in a woman whereas, all NRL-patch tests and patch by patch with a latex gloves resulted negative.

Additional rubber series showed positive results to paraphenilendiamine and thiuram mix allergens in to 2 females.

“TRP-LTX 960” showed positive results in 1 female, that had also displayed a positivity to Thiuram.

We did not perform provocation tests, such as specific nasal provocation and inhalation [18], and use test [19] because there is no standardized use tests material available and for the risk of anaphylactic reactions [20,21]. Furthermore the challenge test does not exclude adverse reactions in the future.

## Discussion

Latex allergy emerged relatively recently as an important medical condition. Until 1979, rubber allergy, especially from gloves, was usually in the form of a type IV delayed hypersensitivity, contact dermatitis reaction [22]. In 1979, Nutter described a woman with type I latex allergy having used household NRL gloves manifested by contact urticaria [23]. With the rapidly increasing number of cases, type I latex allergy became a major medical, occupational health, medico-legal and financial problems during the 1990s [22,24]. By 1997, the US Food and Drug Administration (FDA) had received more than 1700 reports of severe allergic reactions to medical latex devices [25,26]. It is estimated that more than 15 million people suffer from latex allergy worldwide [9-11]. Among the general population the range of sensitization is between 5% and 10%, while in the healthcare workers it is approximately 10 to 17% [12].

Currently there is no agreement about guidelines and patients study protocols and in literature there are no official documents about it and there are discrepancies. In fact, for example, while in Europe and in Canada extracts for latex skin prick test are available [12], in the USA there are no FDA-approved skin test reagents for latex [27]. The only national and international guidelines concerning the prevention and reduction of risk were drawn up in 2008 by an Italian research group [28].

The prevalence of latex allergy depends on the population studied, ranging from 3% to 64%, being highest in certain groups. However in literature there are no data available about latex sensitization in elderly population and this group of patients is not considered among the populations most at risk.

Our study shows that among our elderly population aged 65 or over, 10 patients resulted latex positive, with a prevalence of sensitization of 11,4%, and if compared to the general population of 912 total patients, this percentage decrease to 1% of all patients studied.

However, our results show that allergy to latex is a growing disease that can occur at any age. We therefore suggest to consider people aged over 65 affected by urticaria and angioedema, contact dermatitis, respiratory and/or mucosal reactions, adverse drug reactions and polisensitivity for aeroallergens, as additional risk categories. In addition, elderly patients are more vulnerable because of the frequent presence of comorbidity that often requires hospitalization and surgery. The failure to diagnose such an allergic disease can result in serious consequences. It’s important to identify patients at risk, to make a quick and correct diagnosis and therefore to be able to manage the patient.

So, our proposal is to carry out the study for latex allergy in the following patient-types:

•those aged from 65 to 86, with a peak in the range 75-79 years;

•those reporting diseases such as urticaria, angioedema, adverse drug reactions, contact dermatitis and finally asthma and polisensitivity to aeroallergens;

•we also suggest the submission at the second level in vivo tests (skin prick test) the patients with a highly suspected clinical history for latex sensitivity, even if the RAST is negative.

## Conclusions

The increasing recognition of latex allergy needs a more accurate investigation, starting from the clinical history. In fact, in our study 2% of positive adult patients reported hypersensitivity to latex, but the others had not experienced any problems before retirement. This may be related to more hours spent at home and the hobbies practiced. In fact some of these patients reported having initiated activities such as scuba diving, bowling and stamp collecting. It is important to perform a complete allergological study of these patients to have as much scientific evidences as possible also because in literature there are no studies on latex sensitization in the elderly.

### Consent

Written informed consent was obtained from the patient for the publication of this report and any accompanying images.

## Abbreviations

NRL: Natural rubber latex; ADR: Adverse drug reactions; D2/3/4: Day 2/3/4.

## Competing interests

The authors declare that they have no competing interests.

## Authors’ contributions

TG conceived of the study, and participated in its design and coordination and helped to draft the manuscript. All authors read and approved the final manuscript.
